# Brain-Type Creatine Kinase Release from Cultured Osteoclasts Exposed to Neridronate in Children Affected by Osteogenesis Imperfecta Type 1

**DOI:** 10.3390/biomedicines11020458

**Published:** 2023-02-04

**Authors:** Maria Felicia Faienza, Albina Tummolo, Mauro Celli, Roberto Finocchiaro, Laura Piacente, Francesca Di Serio, Grazia Paola Nicchia, Giacomina Brunetti, Patrizia D’Eufemia

**Affiliations:** 1 Department of Precision and Regenerative Medicine and Ionian area, University of Bari, “A. Moro”, 70124 Bari, Italy; 2 Department of Metabolic Diseases, Clinical Genetics and Diabetology, Giovanni XXIII Children Hospital, Azienda Ospedaliero-Universitaria Consorziale, 70126 Bari, Italy; 3 Department of Pediatric, Sapienza University of Rome, 00185 Rome, Italy; 4 Giovanni XXIII Pediatric Hospital, 70126 Bari, Italy; 5 Clinical Pathology Unit, Azienda Ospedaliero-Universitaria (AOU) Policlinico Consorziale di Bari—Ospedale Giovanni XXIII, 70124 Bari, Italy; 6 Department of Biosciences, Biotechnologies and Environment, University of Bari “A. Moro”, 70125 Bari, Italy

**Keywords:** osteogenesis imperfecta, CK-BB, osteoclastogenesis, neridronate

## Abstract

Brain-type creatine kinase (CK-BB) increases during osteoclastogenesis, with high circulating amounts in type I osteogenesis imperfecta (OI) following treatment with neridronate, a bisphosphonate able to inhibit osteoclast activity and survival. The aim of this study was to demonstrate the correlation between osteoclastogenesis and CK-BB release from OI patients’ osteoclasts treated with different concentrations of neridronate. Our patients showed reduced bone quality, increased levels of CTX I, a marker of bone resorption, and decreased levels of OPG, an inhibitor of osteoclastogenesis. In OI patients, the presence of MCSF and RANKL determined an increased secretion of CK-BB from osteoclasts (*p* = 0.04) compared with control conditions without these cytokines; interestingly, in the absence of these factors, the secretion of CK-BB is significantly elevated at 3 µmol/L compared with 0.03 and 1 µmol/L (*p* = 0.007). In healthy donors’ cultures, the higher concentration of CK-BB can be detected following stimulation with 3 µmol/L neridronate compared with the untreated condition both with and without MCSF and RANKL (*p* = 0.03 and *p* = 0.006, respectively). Consistently, in osteoclast cultures, neridronate treatment is associated with a decrease in multinucleated TRAP+ cells, together with morphology changes typical of apoptosis. Consistently, in the media of the same osteoclast cultures, we demonstrated a significant increase in caspase-3 levels. In conclusion, our findings support the idea that CK-BB levels increase in the serum of OI-treated patients.

## 1. Introduction

Creatine kinase is a dimeric enzyme that catalyzes the reaction of creatine and adenosine triphosphate (ATP) to form phosphocreatine and adenosine diphosphate (ADP), playing an important role in storage and distribution in cellular energetics [1]. There are three isoenzymes: ubiquitous brain-type creatine kinase (CK-BB), sarcomeric muscle type (CK-MM), and cardiac muscle type (CK-MB) [2]. CK-BB is present in a wide range of tissues, including the brain, retina, uterus, testes, and osteoclasts, in which it executes the function of energy maintenance and regulation [3]. Mature osteoclasts resorb bone based on the synthesis and secretion of acid and degradative enzymes while maintaining a high-energy state for active bone resorption [4]. Studies have demonstrated that the CK-BB gene is highly expressed in rabbit osteoclasts [5] and that the receptor activator of nuclear kappa-B ligand (RANKL) up-regulates CK-BB during osteoclastogenesis [6]. Chang et al. [7] reported the CK-BB increase during osteoclastic differentiation of murine bone marrow-derived macrophages cultured in the presence of MCSF and RANKL as well as in RANKL-induced osteoclastogenesis of RAW264.7 cells, thus showing that CK-BB is already expressed on osteoclast precursors. Moreover, the same authors reported that the decrease of CK-BB expression by RNA interference suppresses bone resorption by osteoclasts grown in vitro, showing that CK-BB has a crucial role in bone remodeling [7]. In humans, the elevation of serum levels of CK-BB has been found in some types of osteopetrosis (OPT) in which osteoclasts fail to resorb bone [8,9,10], and in patients in which drug-induced OPT was a consequence of administration of bisphosphonates (BPs) [11]. Moreover, other studies observed an increase of this isoenzyme in the serum of children affected by osteogenesis imperfecta (OI) during treatment with BPs [12,13].

OI is a genetic disorder characterized by increased bone fragility of varying severity, in which osteoblasts produce an abnormal matrix that does not respond to mechanical loads. In compensation, the osteoblast population increases, and osteoclast activity is raised, leading to a high bone turnover rate [14]. There are four main types of OI, and type 1 is the mildest form without major bone deformity. BPs are considered the treatment of choice of OI in the pediatric population, even if some aspects of their complex mechanism of action remain to be elucidated [15]. These drugs inhibit bone resorption by suppressing the activity of osteoclasts and shortening their life span [16]. There is no consensus on criteria to initiate treatment, determine treatment duration, or evaluate the long-term safety, especially in the pediatric population. Many reports have shown CK elevation during BPs treatment, but the mechanism is unclear [11,17]. Tanaka et al. examined CK release induced by amino-BPs from rabbit bone-derived cells in vitro. The result of this study shows that CK release is induced by amino-BPs from osteoclasts and suggests that this phenomenon is an osteoclast apoptosis-related event, providing a possible explanation of the mechanism involved in increased serum CK-BB of patients treated with BPs [18].

In our previous study, we identified a significant increase in serum CK-BB after one year of treatment with amino-BPs (neridronate) in children affected by OI [12]. Since then, serum CK isoenzymes have been investigated in the same children with continuous versus discontinuous neridronate treatment over a further 2-year follow-up period. The results provide evidence of the cumulative effect of neridronate administration in increasing serum CK-BB levels and the reversible effect after its discontinuation. The authors suggest that the evaluation of serum CK-BB could represent a useful biochemical marker in patients receiving prolonged BPs treatment [13]. Although experimental results suggest that serum CK-BB increase during BPs treatment comes from osteoclasts, no studies have evaluated the course of in vitro release from cultured osteoclasts in healthy donors and possible effects of pathology [7,8,9,10]. Thus, we evaluated the CK-BB levels in mature osteoclasts from OI patients cultured without or with the classical pro-osteoclastogenic cytokines treated with different concentrations of neridronate. The same treatment was also performed in cultures taken from healthy donors. The results could provide new insight in order to better evaluate the mechanism of serum CK-BB increase in this bone disease and improve bisphosphonate treatment strategies in this and other pathologies.

## 2. Materials and Methods

### 2.1. Subjects 

Five patients (3 females and 2 males), with a median age of 8.5 (min 5.9–max 18.5), affected by OI type 1 according to the classic Sillence criteria [19] were included in the study. Their anthropometric, clinical, and instrumental data are reported in Table 1. The local ethics committee approved the study. The study was conducted in accordance with the criteria of the declaration of Helsinki. An informed consent form was signed by the patients’ parents. Patients 1 and 2 were siblings, and their father was affected by OI. Patient 1 had a single (tibial) fracture when he was two years old; his sister had one fracture (hand phalanx bone) when she was 4.5 years old. The growth parameters always remained within the normal range in both siblings, who carry the same mutation of the *COL1A1* gene: c.1452delT in exon 21. Patients 3 and 4 were sisters with familial OI in the maternal line: mother, grandmother and her twin, and a maternal cousin. In patient 3, diagnosis was made soon after birth because of bluish sclerae and lower limb dysmetria. Her first fracture of the right tibia occurred at 2 years old. Because of the lower-than-normal growth rate, therapy with GH was commenced and continued until the age of eight years, with recovery of the growth trend. She experienced other fractures at 10 (wrist), 11 (heel), 12 (metatarsal bone) years of age. Patient 4 was diagnosed after birth because of bluish sclerae. She experienced the first fracture at the age of two years (right elbow). Differently from her sister, her growth pattern remained stable at the lower limit of the normal range. She was diagnosed with premature pubarche, at the age of eight, without signs of hormone elevation. Both sisters carry the same gene mutation of *COL1A2* gene: c.13288G>T in exon 13. Patient 5 is of Maghrebian origin with consanguineous parents. After birth, in Morocco, bilateral congenital clubfoot and femoral deformities were noticed, so the family moved to Italy. He underwent a deep diagnostic work-up with the final diagnosis of OI on the basis of *COL1A1* gene mutation: c.4123G>A in exon 50. He suffered two fractures over the follow-up and has never been able to walk unaided. All patients had a daily intake of vitamin D of at least 400 IU/day and of calcium of at least 600 mg/day and underwent specific and individualized physiotherapy. Exclusion criteria were previous treatment with BPs; treatment with drugs that affect bone metabolism (corticosteroids, anticonvulsants, etc.) in the six months prior to the study; acute or chronic gastro-intestinal, cardiovascular, or neuromuscular diseases that induce alterations in serum CK. Four patients had had fractures throughout their life, but no one had presented fractures in the last 12 months prior to the laboratory assessment. As a control group, we enrolled 5 healthy subjects matched by age and sex referred to our hospital for minor surgery or electrocardiographic screening.

### 2.2. Cells and Culture Conditions

Peripheral blood mononuclear cells (PBMCs) were isolated following centrifugation of peripheral blood samples over a Histopaque 1077 density gradient (Sigma Chemical, St. Louis, MO, USA), and cultured in α-MEM (Life Technologies, Paisley, UK) supplemented with 10% fetal bovine serum, 100 μg/mL streptomycin and 100 IU/mL penicillin, (all from Life Technologies, Inc. Ltd., Uxbridge, UK), the PBMCs were cultured in the absence or presence of 25 ng/mL recombinant human MCSF and 30 ng/mL RANKL (both from R&D Systems, Minneapolis, MN, USA) for about 20 days to obtain fully differentiated osteoclasts. Mature osteoclasts were cultured in the absence or presence of neridronate (0.3, 1, and 3 µmol/L—ABIOGEN, Pharma, Pisa) for 48 h, and osteoclasts identified as tartrate-resistant acid phosphatase-positive (TRAP) multinucleated cells (Sigma Aldrich, Milan, Italy) containing three or more nuclei. The concentrations of the neridronate were selected according to literature data [18], even though in the experiments, the authors used minodronic acid, a different amino-BP. However, other authors have explored the effects of other amino-BPs on CK—BB release by osteoclasts, such as risedronate and alendronate [18]. The photomicrographs were obtained using an Eclipse Ti-S microscope (Nikon, Tokyo, Japan) equipped with a Nikon Plan Fluor 10 × l. The microscope was connected to a Nikon digital SIGHT camera. The associated software is Nis-Elements F by Nikon.

### 2.3. Biochemical Assessments

Venous peripheral blood samples were obtained from patients and controls after an overnight fast. CTX-I was assessed by ELISA immunoassay (Serum CrossLaps; Immunodiagnostics Systems, Fountain Hills, AZ, USA). TRACP5b was assessed by immunodiagnostic systems (Boldon Business Park, Boldon, UK, Tyne and Wear, NE35 9PD). The analysis of sRANKL and OPG was performed by using an ELISA kit (Immundiagnostik AG, D-64625 Bensheim, Germany for sRANKL; Biomedica Medizinprodukle GmbH( Wien in Austria, Bensheim, Germany ) and Co KG, A-1210 Wien, Austria for OPG). Intra-assay coefficients of variation for these ELISA assays were <3%.

The levels of CK-BB and caspase-3 were assayed in a culture media. The CK-BB (LSBio, Inc., Seattle, WA, USA) assay range was 1.56–100 ng/mL with intra-assay CV < 10%; inter-assay CV < 12%. 

The caspase-3 (RayBiotech, Norcross, GA, USA) assay range was 0–10,000 pg/mL with an inter-assay CV% < 12% and intra-assay CV% < 10%.

### 2.4. Bone Quality Measurements

The QUS (SONOST 3000; OsteoSys Co., Ltd., Seoul, South Korea) measurement score of the calcaneus region was used to calculate the bone quality index (BQI), which was expressed as a standard deviation score (BQI-SDS) and related to the WHO criteria (normal, osteopenia and osteoporosis) (WHO 2012). The machine was calibrated daily according to the manufacturer’s instructions. The outputs included broadband ultrasound attenuation (BUA, measured in dB/MHz), the bone quality index (BQI), and the speed of sound (SOS, measured in m/s). BUA reproduces bone structure and density by reduction analysis of ultrasound pulse intensity through the bone. SOS states ultrasound wave speed through the bone, thus reproducing bone mineral density. SOS is directly correlated to temperature, while BUA is inversely associated with temperature. These correlation coefficients are combined with SOS and BUA to obtain the BQI (BQI = α × SOS + β × BUA) [20]. Manuals and User Guides for OsteoSys SONOST 3000 are Available online: https://www.manualslib.com/products/Osteosys-Sonost-3000-8710830.html (accessed on 6 November 2022) [20].

### 2.5. Statistical Analyses

Results are reported as means ± standard error. Comparisons between groups were performed by *t*-test. Linear correlations were evaluated with the Spearman correlation coefficient. Sigmaplot version 15.0 (SPSS Inc., Chicago, IL, USA) was utilized for statistical analyses. The limit of statistical significance was set at 0.05.

## 3. Results

### 3.1. Bone Status in OI Patients

Table 1 shows patients’ baseline characteristics as well as biochemical and instrumental parameters of bone metabolism. QUS evaluation showed that only patient 4 showed a normal BQI-SDS, whereas the other patients showed a reduction in BQI-SDS. Consistently, with these data, all patients displayed lower OPG levels compared with the physiological range, suggesting greater osteoclast activity. However, sRANKL levels fall in the normal range. These data are supported by the increased levels of CTX I observed in most enrolled patients, whereas TRAcP5b values fell in the normal range. Interestingly, we found that BQI-SDS is inversely related to CTX I (r = −0.975, *p* < 0.01) and sRANKL (r = −0.975, *p* < 0.01).

### 3.2. CK-BB Levels in Osteoclast Culture from OI Patients and Controls

**Figure 1:**
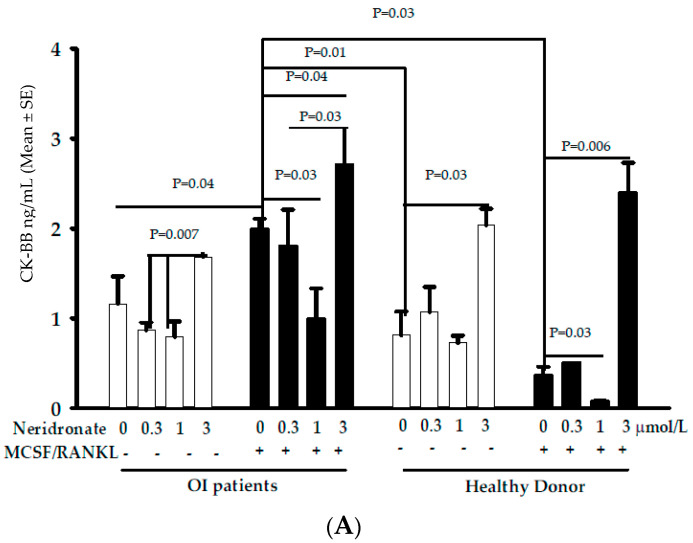

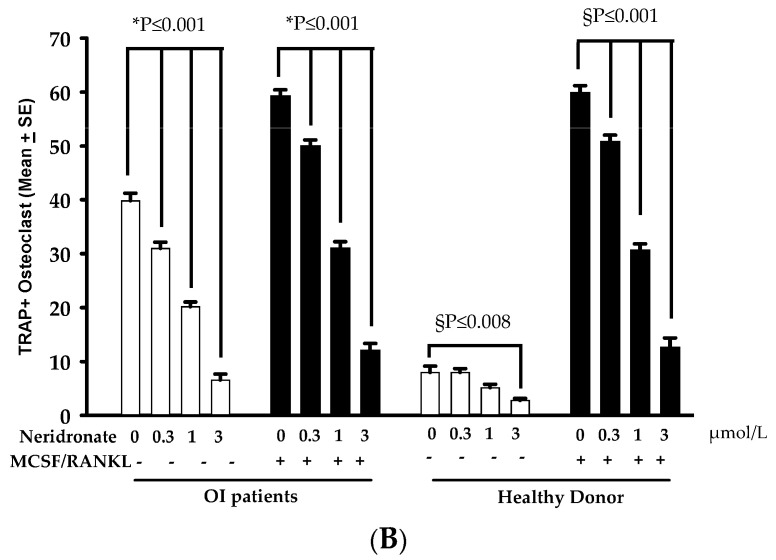
(**A**) CK-BB levels in media of osteoclast cultures and (**B**) number of TRAP+ osteoclasts from a representative OI patient and a representative healthy donor treated with different concentrations of neridronate. Each treatment was performed in quintuplicate replication, and each bar reports the results as mean ± SE. * and § are referred to as 0 neridronate.

**Figure 2:**
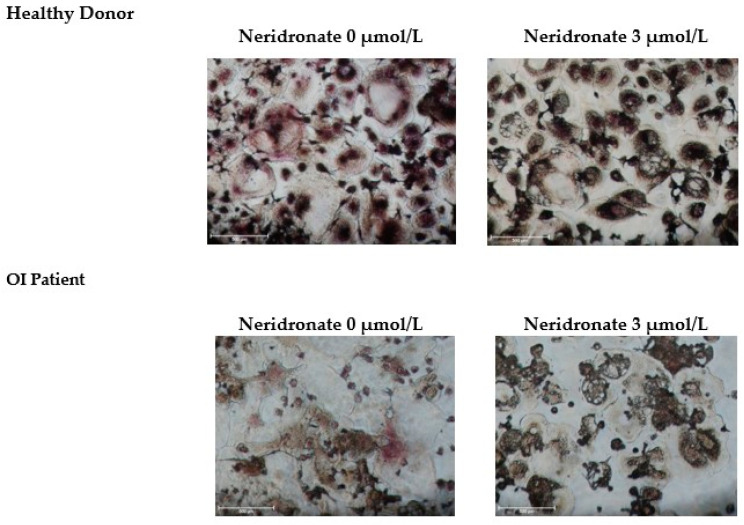
Multinucleated TRAP+ osteoclast cultures from a representative healthy donor and OI patient treated with MCSF and RANKL without or with 3 µmol/L neridronate. Bars: 500 µm.

Altered osteoclastogenesis has been reported in OI [22], and osteoclasts represent an important source of CK-BB. Thus, -PBMCs from healthy donors and OI patients without or with MCSF and RANKL were cultured for about 20 days of cultures (when mature osteoclasts appeared in cultures from patients in both conditions and in cultures from healthy donors in the presence of the pro-osteoclastogenic cytokines). At this time, cultures were treated with different concentrations of neridronate (48 h) in the absence or presence of MCSF and RANKL. After these treatments, media were collected and used for ELISA (Figure 1A). Our results show that in OI patients in the absence of these pro-osteoclastogenic factors, the secretion of CK-BB is significantly elevated at 3 µmol/L of neridronate compared with 0.3 and 1 µmol/L neridronate (*p* = 0.007); however, the differences were not statistically significant compared with the untreated condition. Interestingly, the presence of MCSF and RANKL determined an increased secretion of CK-BB (*p* = 0.04) compared with the control condition without these cytokines. In OI patients’ cultures with MCSF and RANKL, a double effect can be observed: there is a significant decrease in CK-BB levels at 1 µmol/L neridronate compared with the control condition without neridronate (*p* = 0.03), whereas at 3 µmol/L neridronate the highest levels of CK-BB can be detected with respect to the control without neridronate (*p* < 0.04) and to 1 µmol/L neridronate (*p* = 0.03). Interestingly, the levels of CK-BB are higher in OI cultures in the absence or presence of MCSF and RANKL with respect to the healthy donor (*p* = 0.01 and *p* = 0.03, respectively). In healthy donor’s cultures the higher concentration of CK-BB could be detected following stimulation with 3 µmol/L neridronate compared with the untreated neridronate condition both in the absence or presence of MCSF/RANKL (*p*= 0.03 and *p* = 0.006, respectively). As observed in cultures from OI patients and in cultures from healthy donors in the presence of the pro-osteoclastogenic cytokines, the treatment with 1 µmol/L neridronate determined a significant decrease in the CK-BB secretion compared with the control condition untreated with neridronate (*p* < 0.03). Thus, confirming a potential double effect of neridronate on osteoclast cultures at low and high concentrations, respectively. Consistently, looking at osteoclast cultures stained with TRAP, a statistically significant dose-dependent decrease in multinucleated cells can be counted (Figure 1B). This condition is particularly evident for the OI patients in which osteoclastogenesis also occurred in the absence of MCSF and RANKL. The strongest effect on differentiation is evident at 3 µmol/L neridronate, which is the condition associated with the highest levels of CK-BB in the media. It seems that at low concentrations, neridronate inhibited the differentiation of the multinucleated cells, whereas at 3 µmol/L neridronate induced apoptosis. In fact, the cells in this latter condition lost the classical shape, and some of them also died before staining (Figure 2). 

### 3.3. Caspase-3 Mediated Apoptosis in Neridronate-Treated Cultures

**Figure 3:**
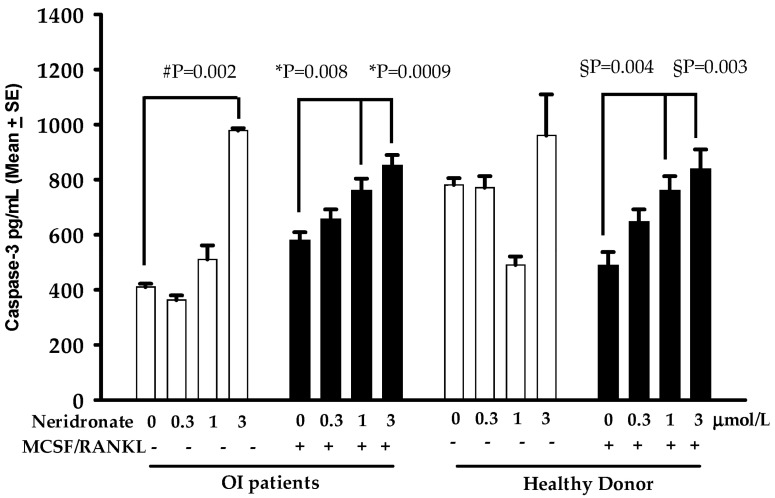
Caspase-3 levels in media of osteoclast cultures from a representative OI patient and a representative healthy donor treated with different concentrations of neridronate. Each treatment was performed in quintuplicate replication, and each bar reports the results as mean ± SE. #, * and § are referred to as 0 neridronate.

The reported results prompted us to focus on the pro-apoptotic effect of neridronate on osteoclast apoptosis by measuring the levels of caspase-3 in the same media used to detect CK-BB. Caspase-3 is an effector caspase; thus, measuring its amount can be used as a guarantee of the active apoptotic process. Interestingly, we found that, in the media, the levels of caspase-3 progressively increased following neridronate treatment, reaching the highest levels in cells treated with 3 µmol/L neridronate (Figure 3). In detail, caspase-3 levels are significantly higher in the osteoclast cultures of OI patients cultured without MCSF and RANKL but treated with 3 µmol/L neridronate (*p* = 0.002). The presence of the pro-osteoclastogenic cytokines in the OI patients’ cultures is associated with an increase in the release of caspase-3 in the culture media following the treatment with 1 and 3 µmol/L neridronate (*p* = 0.008 and *p* = 0.0009, respectively). Very interestingly, in the media of cultures taken from healthy donors, in the treatment with neridronate, the caspase-3 levels displayed a tendency to increase but did not reach statistical significance. Conversely, a similar behavior to OI patients’ cultures can be observed in osteoclast cultures taken from healthy donors treated with neridronate. In particular, the levels of caspase-3 are significantly increased in healthy donors’ osteoclast cultures simultaneously treated with MCSF and RANKL together with 1 and 3 µmol/L neridronate (*p* = 0.004 and *p* = 0.003, respectively).

## 4. Discussion

The results of this study support the altered activity of osteoclasts in OI, as demonstrated by the high levels of CTX I and reduced amounts of OPG, with consequent alteration of bone quality. Furthermore, we have also shown that the treatment in vitro of mature osteoclasts with neridronate, a bisphosphonate currently used for OI management, leads to the increased production of CK-BB together with a decrease in osteoclast number that is linked to the caspase-3 release due to the activation of the apoptotic process.

The altered bone quality is a characteristic of OI and is associated with all bone cell dysregulation, thus influencing each other. Consistently, we have demonstrated that the reduced BQI-SDS in 4 out of 5 patients is also inversely related to sRANKL and CTX I. It is important to remember that one of the major sources of RANKL and OPG is osteoblasts, and we have previously reported that the sera of OI patients in vitro can inhibit osteoblast differentiation as well as reduce OPG secretion without altering RANKL production [22], thus, supporting our findings in the present paper. Immune cells [23,24] also contribute to the synthesis of these cytokines, and interestingly, we previously detected the reduced expression of OPG in peripheral blood mononuclear cells of OI patients, both treated or not with bisphosphonates. Li et al. report that the altered RANKL/OPG is linked to a more immature phenotype of osteoblasts in OI murine model [25].

Additionally, the altered bone quality, together with the increased activity of osteoclasts in OI, led to the identification of BPs as the gold standard therapy for its management, also in pediatric-age patients, where neridronate is the most used BP. These drugs can act on osteoclasts by suppressing their resorption activity as well as by shortening their lifespan [16]. However, the treatment of OI type I with bisphosphonates is associated with many doubts as to the dose to be used or safety, especially in children. Long-term inhibition of osteoclasts by bisphosphonates may reduce bone quality, leading to non-dynamic bones where micro-damage is not repaired and accumulates, possibly causing an overall increase in bone fragility [26].

Amino-BPs are characterized by two phosphate side chains on the central carbon atom in the P–C–P backbone that are necessary for bone binding with complementary interactions through a hydroxyl group [27,28]. Amino-BPs also have a nitrogen-containing side chain on the central carbon atom that leads to the inhibitory strength for farnesyl pyrophosphate (FPP) synthase in the mevalonate pathway. Consequently, the side chains lead to antiresorptive activity [29]. Bone-bound amino-BPs are internalized by osteoclasts through bone resorption, thus reducing their activity or viability through the inhibition of FPP synthase. Amino-BP antiresorptive effect involves the prenylation of small G proteins like Rab [30,31], while the pro-apoptotic effect involves the activation of the ERK/Bim axis [32]. Bone-bound amino-BPs are liberated by the acidic microenvironment created by the same osteoclasts during bone resorption and thus endocytose themselves. These kinds of osteoclasts are not active in bone resorption and prone to apoptosis. Different papers demonstrate the activation of the apoptotic pathway following the treatment of osteoclasts with bisphosphonates through caspase-3 involvement. In particular, Tai et al. report that bisphosphonate induces cell apoptosis with morphological changes in human and murine osteoclast precursors and mature osteoclast-like cells via caspase-3 activation [33]. 

It has been reported that N-BPs led to CK-BB release from osteoclasts, and it has been hypothesized that CK-BB is just released from damaged osteoclasts into extracellular fluid following N-BP treatment. Consistently, the serum levels of CK-BB are augmented in patients treated with N-BPs, and the levels of CK-BB in sera can represent a suitable marker for osteoclast alteration due to N-BP treatment. In detail, D’Eufemia et al. reported that CK-BB levels time-dependently increased in OI patients treated with neridronate, whereas its levels were slightly but not significantly elevated with respect to the controls before starting treatment [12]. The possible reason for the CK-BB increase in the serum of OI-treated patients can be explained by remembering that CK-BB is fundamental for osteoclast activity [7]. In detail, CK-BB has a crucial role in osteoclast function through indirect or direct regulation of Rho and V-ATPase [7]. These findings suggested that it is important inside the cells; thus, the fact that its concentration increases following BP treatment can be explained by the release of CK-BB in the bone microenvironment and, thus, in sera following osteoclast apoptosis.

An important aspect of this study is the evaluation of in vitro exposure to different neridronate concentrations on CK-BB release both from cultured osteoclasts of OI patients and healthy donors. A significantly increased secretion of CK-BB in OI patients and controls is observed with an exposure of osteoclasts to a neridronate concentration of 3 µmol/L. The addition of pro-osteoclastogenic factors to the culture medium enhanced this phenomenon. Interestingly, exposure to lower concentrations of neridronate (1 µmol/L) determines a significant decrease in CK-BB release with respect to the basal condition in both study groups. It could appear strange that healthy donors’ cultures without MCSF and RANKL, displaying very few osteoclasts secreted a similar amount of CK-BB, respect the same cultures of OI patients, but the literature data support our data. In particular, Chang et al. [7] reported the CK-BB increase during osteoclastic differentiation of murine bone marrow-derived macrophages cultured in the presence of MCSF and RANKL as well as in RANKL-induced osteoclastogenesis of RAW264.7 cells, thus showing that CK-BB is already expressed on osteoclast precursors and reached the maximum levels in completely differentiated osteoclasts. Other differences in CK-BB secretion by osteoclasts from OI patients and controls could be explained by the different microenvironments that can be created in the cultures. In fact, as reported in our previous work [22], PBMCs from OI patients are quite different from healthy donors, with the former characterized by the production of pro-inflammatory cytokines and a different phenotype compared with the latter.

Additionally, in this case, the addition of pro-osteoclastogenic cytokines enhanced the effect of neridronate on CK-BB release. These findings suggest a potential double effect of neridronate on osteoclast cultures at low or high concentrations. In fact, the cells in this condition lost their classical shape, and some cells also died before staining. Consistently, we demonstrated that the treatment with neridronate determined in the media of osteoclast cultures the increase of caspase-3 levels for patients in cultures both treated or not MCSF and RANKL, whereas in healthy donors only in cultures with the pro-osteoclastogenic cytokines. The difference between patients and healthy donors could be explained by the few osteoclasts that differentiated without MCSF and RANKL in the cultures of the latter, although the trend appeared without reaching statistical significance. Furthermore, we cannot exclude that the caspase-3 levels detected in cultures derived from PBMCs of healthy donors without pro-osteoclastogenic cytokines derived by the precursors that died for the lack of MCSF and RANKL.

Since OI type I represents a genetic alteration of collagen synthesis, a protein not expressed in osteoclasts, the cause of this interesting finding should be sought in the altered biochemical environment that, in vivo, the osteoclasts of OI patients suffer due to the specific derangement of the bone metabolism of OI. The altered activity of osteoclasts in OI has been demonstrated in bone biopsies as well as in animal models [34,35,36,37]. Sistermans et al. [38] report that in bone sections, osteoclasts are positive for CK-BB following immunohistochemical staining but not osteocytes. Osteoblasts have been reported to express CK-BB, and its levels are modulated by estrogens [39]. Vascular endothelial cells also expressed CK-BB [40]. As osteoblasts, endothelial cells can express CK-BB; thus, we cannot exclude that, in vivo, they contribute to determining the serum levels of CK-BB. Consistently, Chang et al. [7] demonstrated that CK-BB deficient mice did not display a different bone phenotype with respect to the wild-type mice, although in vitro osteoclasts from CK-BB deficient mice are unable to resorb bone compared with those from wild-type mice. Differently, the same deficient mice are protected against bone loss associated with ovariectomy and lipopolysaccharide treatment [7].

The fact that OI osteoclasts maintain an altered response to cytokines even when studied in vitro conditions is an intriguing finding deserving of further investigation. A possible explanation could involve epigenetic changes in the osteoclasts of OI patients leading to an apoptotic trend after pro-osteoclastogenic cytokine stimuli.

The strength of the study is the performance of the experiments without the pro-osteoclastogenic cytokines MCSF and RANKL on human samples of untreated OI pediatric patients using different concentrations of neridronate.

The paper presents some weaknesses, such as the use of QUS instead of DXA, the small size sample, together with the lack of a resorption assay. Although DXA represents the gold standard method for the evaluation of bone mineral density, in pediatric-age patients, QUS is preferred due to the low radiation necessary to obtain information on bone quality. Publications recommend its use; moreover, the altered bone structure in OI is associated with deformities and fractures detected using X-ray [19]. The number of patients enrolled in the study could appear small; this is due to the selection of untreated OI pediatric patients, together with the necessity of working on fresh blood samples for osteoclast cultures.

The pediatric age, together with the ethics restrictions, made it necessary to work with small-volume blood samples; thus, we only use it to plate cells without MCSF/RANKL with replicates, without performing a resorption assay. However, in our previous study [22], even though not reported, we performed the resorption assay, and we found that osteoclasts obtained without and with MCSF/RANKL both resorb bone. It is also important to remember that we performed the neridronate treatment on mature osteoclasts for 48 h; thus, to evaluate the effect on bone resorption, we would have had to detach osteoclasts to be plated on opportune plates to evaluate their resorption activity: this is not possible, because osteoclasts are big cells and treatment with detaching agents damages osteoclasts. Furthermore, the anti-resorptive activity of BPs is widely reported in the scientific literature.

Consistently, our experiments show a significant increase in CK-BB in the media of osteoclast cultures at the highest levels used concentration of neridronate, and its levels are much more elevated in the presence of MCSF and RANKL. To this extent, it has been reported that RANKL treatment increases CK-BB levels during osteoclastogenesis [6]. 

## 5. Conclusions

In conclusion, our findings support the idea that CK-BB levels increase in the serum of OI-treated patients through its secretion by osteoclasts that underwent apoptosis; however, a small amount deriving from osteoclast precursors could not be excluded.

## Figures and Tables

**Table 1:** Anthropometric, clinical, and quantitative ultrasound data of the study population.

	Patient 1	Patient 2	Patient 3	Patient 4	Patient 5
Gender	M	F	F	F	M
Age (years)	8.5	5.9	14.2	18.5	7.5
Height SDS	1.71	3.04	−2.32	−2.11	−0.93
Weight SDS	0.95	1.98	0.27	−0.76	1.22
BMI SDS	0.07	0.89	1.27	0.37	1.97
Tanner stage [21]	I	I	IV	V	I
Blu sclera (Yes/no)	no	yes	yes	yes	yes
Dentinogenesis imperfecta (yes/no)	no	no	yes	no	yes
Number of fractures	1	1	4	1	2
Scoliosis (yes/no)	no	no	yes	no	yes
Limited mobility (yes/no)	no	no	no	no	yes
Leg deformities (yes/no)	Tibial valgus	Tibial valgus	Lower limb dysmetria (left > right)	No	Coxa and femoral valgus; bilateral clubfoot; lower limb dysmetria (right > left)
QUS parameters					
BUA (dB/MHz)	81	36.1	56.2	117.6	34.8
SOS (m/s)	1486.2	1477.2	1478.8	1540	1490.3
BQI SDS	−1.6	−2.6	−3.5	0.1	−2.6
CTX-I (0.034–0.635 ng/mL)	1.2	1.5	0.293	0.335	1.308
TRAcP5b (1.2–4.8 U/L)	2.6	2.3	2.8	2.4	2.1
sRANKL (<1000 pmol/L)	36.4	48.7	4	32.4	87.2
OPG (4.37–5.03 pmol/L)	3.15	3.21	4.14	2.82	1.89

Reference values for the healthy population are reported in brackets. SDS: Standard Deviation Score.

## Data Availability

Data is unavailable due to privacy or ethical restrictions.
